# Derivation of a Nonstoichiometric 1/1 Quasicrystal Approximant from a Stoichiometric 2/1 Quasicrystal Approximant and Maximization of the Magnetocaloric Effect

**DOI:** 10.1021/jacs.5c05947

**Published:** 2025-08-27

**Authors:** Farid Labib, Hiroyuki Takakura, Asuka Ishikawa, Takenori Fujii, Ryuji Tamura

**Affiliations:** † Research Institute of Science and Technology, 26413 Tokyo University of Science, Tokyo 125-8585, Japan; ‡ Division of Applied Physics, Faculty of Engineering, 83969 Hokkaido University, Sapporo 060-8628, Japan; § Cryogenic Research Center, 13143 The University of Tokyo, Bunkyo, Tokyo 113-0032, Japan; ∥ Department of Materials Science and Technology, 26413 Tokyo University of Science, Tokyo 125-8585, Japan

## Abstract

The present research introduces a novel strategy for tuning magnetic properties by overcoming the compositional limitation of stoichiometric intermetallic compounds via extension of their compositional domain into the valence electron-per-atom (*e/a*) parameter space. Focusing on approximant crystals (ACs), a “double heterovalent elemental substitution” is employed in a stoichiometric Ga–Pt–Gd 2/1 AC whereby *e/a* is lowered from approximately 1.98 to 1.60. Through this approach, a new family of Ga-based Tsai-type 1/1 ACs with an exceptionally wide compositional domain within *e/a* space is derived. Remarkably, the magnetic ground state is altered from initially spin-glass to ferromagnetic (FM) with second-order phase transition and mean-field-like critical behavior. More importantly, through this strategy, the isothermal magnetic entropy change (*ΔS*_M_) enhanced significantly and reached a maximum value of −8.7 J/K mol-Gd under a 5 T magnetic field change, even comparable to leading rare-earth magnetocaloric materials including RCo_2_ phases. These findings demonstrate the high potential of a double heterovalent elemental substitution for tailoring magnetic properties and magnetocaloric response in stoichiometric compounds, offering a new pathway for designing high-performance magnetic refrigeration materials even beyond the quasicrystals and ACs.

## Graphical abstract

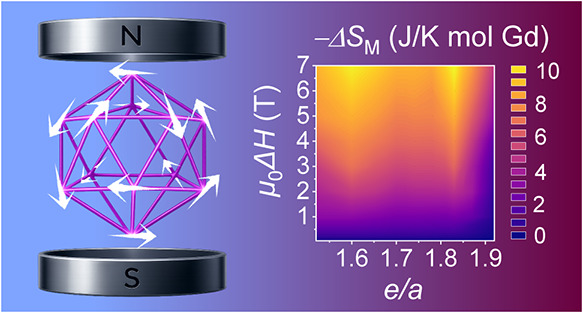

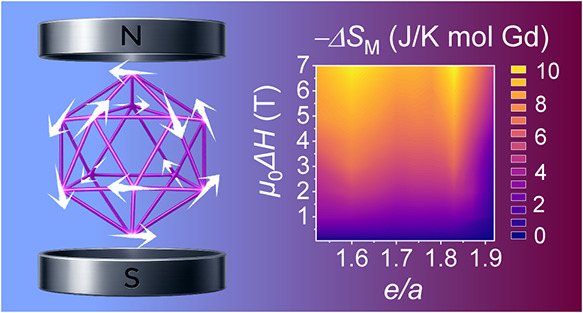

## Introduction

The number of valence electrons per atom (*e/a*) has long been considered a critical parameter governing the electronic structure and stability of intermetallic compounds, as certain structures such as body-centered cubic, complex cubic lattices including γ-brass phases, hexagonal close-packed phases, and quasicrystals (QCs) are known to be stabilized at particular *e/a* values (≈1.5, ≈1.62, ≈1.75, ≈2.00, respectively).^1,2^ Historically, the influence of *e/a* on magnetic properties has been discussed through the Slater–Pauling principle,^3^ which relates the magnetic moment to the number of valence electrons.

In recent years, such an *e/a*-based design principle has played a crucial role in controlling the magnetic properties of a certain class of complex alloy systems. A notable example includes the *nonstoichiometric* Au–SM–R (SM: semimetal, R: rare-earth) Tsai-type 1/1 approximant crystals (ACs), wherein the Au/SM ratio can be varied over a wide range of ∼33 at. % without affecting the underlying crystallographic symmetry.^4^ Reducing *e/a* ratio in these 1/1 ACs has been shown to induce long-range ferromagnetic (FM)^5–8^ and antiferromagnetic (AFM)^9–12^ orders with intriguing non-coplanar whirling spin structures.^13,14^ More recently, the emergence of FM^15,16^ and AFM^17^ orders has also been evidenced in real p-type icosahedral QCs.

While remarkable achievements have been made in controlling magnetic behavior via *e/a*-tuning, one of the modern challenges in the study of intermetallic compounds, including but not limited to QCs and ACs, remains unaddressed. The challenge is that the inherent stoichiometry of many alloys severely limits degrees of freedom in compositional and consequently *e/a* tuning, which is essential for tailoring magnetic properties. Many known QCs and ACs, for instance, are stable near an *e/a* ≈ 2.00 ^2^ and often exhibit spin-glass behavior as a result of geometric frustration and competing interactions, as in Zn-Mg-*R* ^18–20^ and Cd-Mg-*R* ^21–26^ iQCs/ACs.

In the present work, a novel materials design strategy is introduced to overcome such a limitation by extending the compositional domain of stoichiometric compounds in *e/a* parameter space, thereby enabling magnetic property tuning. A central focus of the present work is on enhancing magnetic properties including magnetic entropy response via tailoring the *e/a* parameter. Following the approach introduced in this work, called “*double hetero-valent elemental substitution*”, we report development of an entirely new family of Tsai-type FM Ga-Pt-Au-Gd 1/1 ACs from a parent stoichiometric Ga_52_Pt_34_Gd_14_ 2/1 AC^27^ with a spin-glass-like behavior.

The resultant quaternary 1/1 ACs are all heat treated at 1073 K (corresponding to more than 89% of their melting temperature of ∼1203 K) for a long duration (50 h) to achieve an equilibrium state. They exhibit a second-order FM transition following mean-field critical behavior near the Curie temperature (*T*_C_) across a wide *e/a* space. Through this strategy, *e/a* decreases to ≈1.60 from an initial value of ≈1.98. Following such reduction in *e/a*, the isothermal magnetic entropy change (*ΔS*_M_) enhanced significantly and reached a maximum value of – 8.7 J/K mol-Gd under an applied field change of 5 T, which is outstanding not only among QCs and ACs but also among other magnetocaloric materials. These results suggest *e/a* tuning as a robust and effective strategy to gain unprecedented control over the magnetocaloric effect of the intermetallic compounds. The approach taken in the present work is, in principle, applicable to other alloy systems upon several considerations that will be discussed later, thus may be regarded as a universal tuning strategy.

This paper has been structured into four sections dealing with 1) phase and structure characterization, 2) magnetic properties, 3) critical behavior near *T*_C_, and 4) magnetocaloric effect.

## Results

### 1 Phase and Structure Characterization

In this work, the stoichiometric Ga_52_Pt_34_Gd_14_ 2/1 AC is selected as a parent compound for a double heterovalent elemental substitution, whereby Ga and Pt are partially exchanged with Au. The nominal compositions of the resultant polycrystalline Ga-Pt-Au-Gd 1/1 ACs are listed in Table 1. The elemental substitution performed here reduced the order of AC from 2/1 to 1/1, thereby relaxing the composition constraints, which resulted in a significant expansion of the compositional domain. This is evident from the results of the Le-Bail fittings of powder X-ray diffraction (XRD) patterns shown in Figure 1. The fittings were performed using the JANA 2020 software suite,^28^ assuming space group *Pa*3̅ for the Ga_52_Pt_34_Gd_14_ 2/1 AC parent alloy and *Im*3̅ for the resultant quaternary 1/1 ACs. The excellent agreement between the calculated and observed patterns evidenced by satisfactory fitting parameters confirms the high structural quality of the synthesized samples. It also indicates that the resultant quaternary 1/1 ACs are essentially isostructural in their crystallographic symmetry. Notably, the structure of 1/1 AC accommodates up to 35 atom % Au without affecting the underlying crystallographic symmetry, which is exceptional among many intermetallic compounds. To verify assumed crystallographic symmetries in the Le Bail fittings, a single-crystal X-ray diffraction (SCXRD) experiment was carried out on a representative 1/1 AC with a nominal composition Ga_33_Au_33_Pt_20_Gd_14_. The constructed reciprocal-space sections perpendicular to the [100], [110], and [111] crystallographic axes (as shown in Figure S1­(a–c), respectively) display no violation of the systematic extinction rule for the space group *Im*3̅, confirming the Le Bail fitting results.

**Table 1:** Nominal Compositions, AC Order, Electron per Atom (*e*/*a*), and Magnetic Properties of the Ga-Pt-Au-Gd Compounds^a^

Composition	AC type	*e/a*	Magnetic state
Ga_52_Pt_34_Gd_14_	2/1AC	1.98(1)	SG
Ga_46_Au_7_Pt_33_Gd_14_	1/1AC	1.87(1)	SG
Ga_43_Au_12.5_Pt_30.5_Gd_14_	1/1AC	1.84(1)	SG/FM
Ga_40_Au_21_Pt_25_Gd_14_	1/1AC	1.83(1)	FM
Ga_39_Au_22_Pt_25_Gd_14_	1/1AC	1.81(1)	FM
Ga_37_Au_25_Pt_24_Gd_14_	1/1AC	1.78(1)	FM
Ga_33_Au_33_Pt_20_Gd_14_	1/1AC	1.74(1)	FM
Ga_33_Au_31_Pt_22_Gd_14_	1/1AC	1.72(1)	FM
Ga_31_Au_35_Pt_20_Gd_14_	1/1AC	1.70(1)	FM
Ga_30_Au_33_Pt_23_Gd_14_	1/1 AC	1.65(1)	FM
Ga_28_Au_33_Pt_25_Gd_14_	1/1 AC	1.60(1)	FM
Ga_25_Au_35_Pt_26_Gd_14_	1/1 AC	1.52(1)	SG

a Considering the weighing precision and possible variations during arc melting, the uncertainty in atomic fraction is estimated to be within ±0.5 at. % per element. This corresponds to an uncertainty in *e/a* of approximately ±0.01.

**Figure 1:**
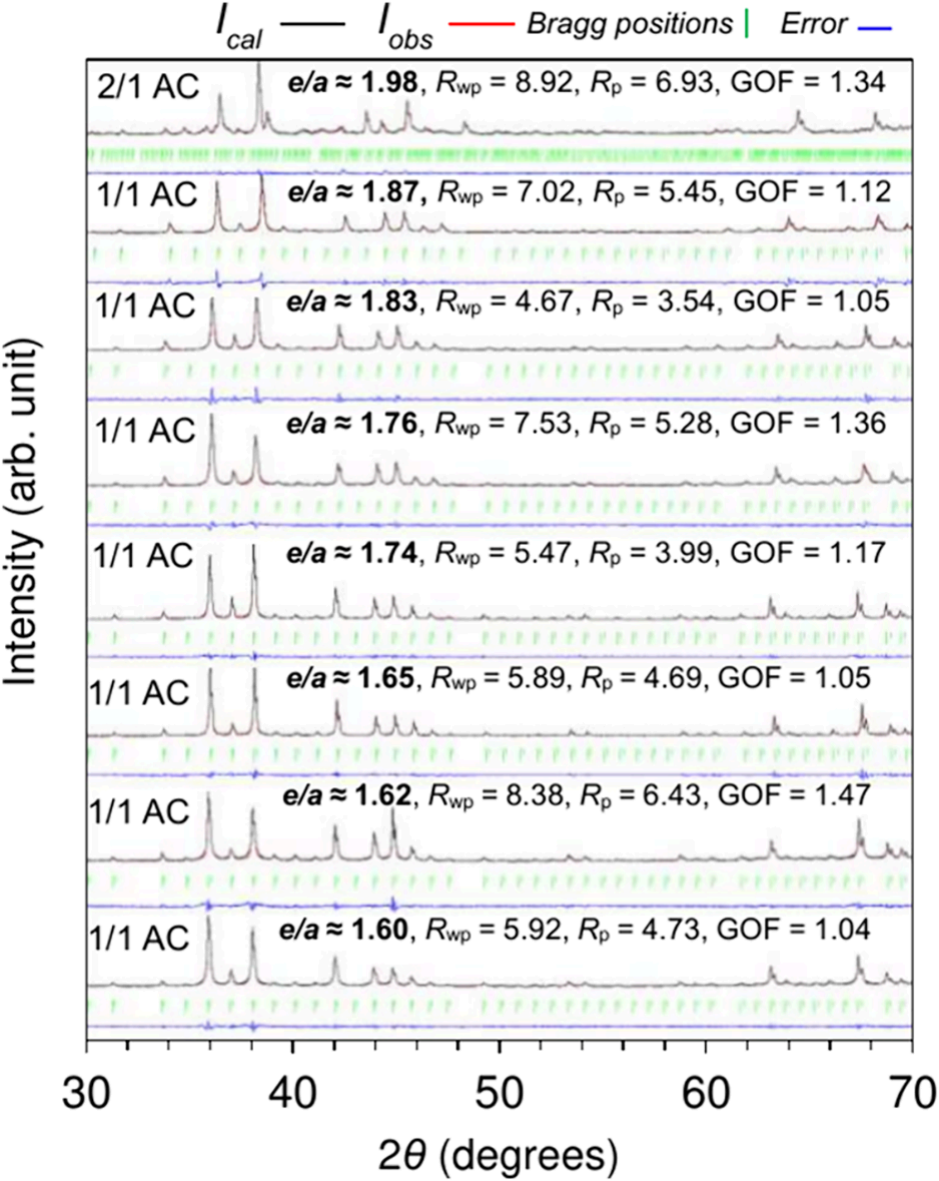
Results of the Le Bail fitting of powder X-ray diffraction (XRD) patterns of the Ga-Pt-Au-Gd ACs annealed at 1073 K. The calculated (*I*_cal_) and measured (*I*_obs_) intensities and their difference are represented by red, black, and blue, respectively, while the green vertical bars indicate the expected Bragg peak positions. The weighted-profile *R*-factor (*R*_wp_), the profile *R*-factor (*R*_p_), and the goodness-of-fit (GOF) for each fit are provided inside each panel.

Given that the stability of 1/1 and 2/1 ACs as well as QCs is often energetically competitive, the switch of AC order from 2/1 to 1/1 upon Au substitution (see Figure 1) suggests a change in free energy landscape to take place in favor of the 1/1 AC. The more important impact of Au substitution is the decrease of *e/a* from ≈1.98 to ≈1.60. Such *e/a* reduction influences the magnetic exchange interactions and the magnetocaloric effect, as will be discussed later. For estimating *e/a*, Mizutani’s scale^29^ has been adopted, which assigns electron valences as Ga = 3, Pt = 0, Au = 1, and Gd = 3. For instance, an *e/a* of Ga_52_Pt_34_Gd_14_ becomes [(52 × 3) + (34 × 0) + (14 × 3)]/100 = 1.98. An uncertainty of ±0.5 at. % in the nominal atomic percentage of each element is considered, which corresponds to an uncertainty of approximately ±0.01 in the estimated *e/a* values.

### 2 Magnetic Properties

The inverse magnetic susceptibility (*H*/*M*) of the samples under μ_0_ *H* = 0.1 T within a temperature range of 1.8–300 K (shown in Figure S2 in the Supporting Information) demonstrates a linear behavior fitting well to the Curie–Weiss law: χ­(*T*) = *N*_A_μ_eff_^2^μ_B_^2^/3*k*_B_(*T* – θ_*w*_) + χ_0_, where *N*_A_, μ_eff_, μ_B_, *k*_B_, θ_w_, and χ_0_ denote the Avogadro number, effective magnetic moment, Bohr magneton, Boltzmann constant, Curie–Weiss temperature, and the temperature-independent magnetic susceptibility, respectively. By extrapolating linear least-squares fittings within a temperature range of 50–300 K, θ_w_ within a range of −10.47(35) to +14.23(41) K is derived. This leads to μ_eff_ values in a range of 7.85(3)–8.12(3) μ_B_, close to the calculated value for free Gd^3+^ ions defined by *g*_*J*_(*J*(*J* + 1))^0.5^ μ_B_ = 7.94 μ_B_,^30^ indicating localization of the magnetic moments on Gd^3+^ ions. The uncertainties in the θ_w_ and μ_eff_ values correspond to standard deviations in the linear fits to the data over different temperature ranges. A polynomial fitting of θ_w_/*dG* (*dG* denotes de Gennes parameter) versus *e/a* in the inset of Figure S2 of the Supporting Information evidences a sharp rise below *e/a* ≈ 1.9 followed by a mild fall below *e/a* ≈ 1.70. This indicates enhanced FM interactions due to the Au contribution reaching a maximum around *e/a* ≈ 1.70 but weakening below that.

Change in magnetic interactions upon *e/a* tuning can be better observed from the temperature dependence of zero-field-cooled (ZFC) and field-cooled (FC) dc magnetic susceptibility (*M*/*H*) shown in Figure 2. Clearly, by reducing the *e/a* ratio, the magnetic response enhances, and plateaus appear in magnetic susceptibilities. The appearance of a plateau is a common feature of Gd-based FM QCs and ACs (see for example refs 5, 15, 31, and 32). Since the *M*–*T* curves in Figure 2 are measured under a very low magnetic field of 0.01 T, the appearance of such a plateau indicates that the slope of the field dependence magnetization (*M* vs *H*) curves near the origin (Figure 3) is temperature independent. The magnetization in this low-field region is likely governed by the magnetic domain wall motion. Nevertheless, the origin of this plateau remains an intriguing issue that needs to be elucidated in future studies.

**Figure 2:**
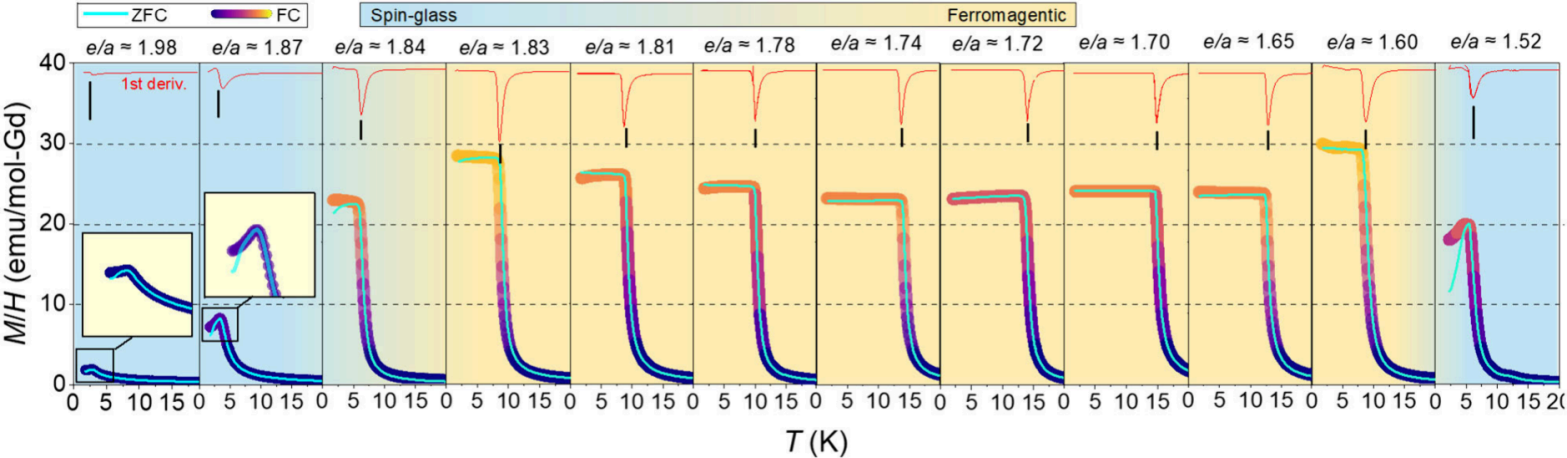
Zero-field-cooled (ZFC) and field-cooled (FC) dc magnetic susceptibility (*M*/*H*) curves of the synthesized samples with various *e/a* values within 1.8 < *T* < 20 K measured under μ_0_Δ*H* = 0.01 T.

**Figure 3:**
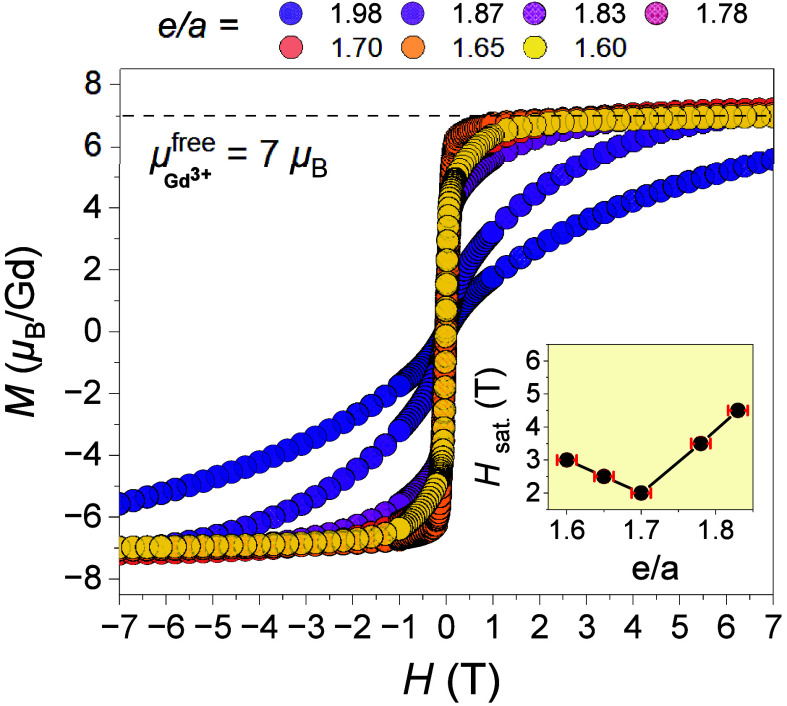
Field dependence of magnetization for selective ACs with various *e/a* ratios up to 7 T. The inset shows the variation of the saturation field *H*_sat._ with *e/a*, where the error bars represent the uncertainties in both *e/a* values (±0.01) and *H*_sat._ values derived from *M*–*H* curves.

In addition, aligned with the trend observed in θ_w_, by reducing the *e/a*, the *T*_C_ rises, reaching 14.91(4) K at *e/a* ≈ 1.70 followed by its subsequent drop at lower *e/a*. The *e/a* dependence of spin dynamics is further captured through ac magnetic susceptibility measurement under frequencies spanning 3 orders of magnitude from 0.1 to 100 Hz, as shown in Figure S3 of the Supporting Information. A frequency dependency in the in-phase component of ac magnetic susceptibility (χ′_ac_) is only observed in Ga_52_Pt_34_Gd_14_ 2/1 AC, Ga_46_Au_7_Pt_33_Gd_14_ 1/1 AC, Ga_25_Au_35_Pt_26_Gd_14_ 1/1 AC, and to lesser extent in the Ga_43_Au_12.5_Pt_30.5_Gd_14_ 1/1 AC, indicating the presence of a metastable component associated with spin-glass-like behavior in spin-glass regions of the *e/a* parameter space. Based on ac susceptibility results, spin-glass and FM regions are distinguished by different background colors in Figure 2.

To further verify establishment of FM order, *M* vs *H* of selective ACs with varying *e/a* ratios is measured (Figure 3). Clearly, the *M* of all quaternary 1/1 ACs with *e/a* range within 1.60(1)–1.83(1) reaches a full moment of a Gd^3+^ free ion based on Hund’s rule (i.e., 7.00 μ_B_/Gd^3+^), though the saturation field (*H*_sat._; a field above which the *M* reaches 7 μ_B_/Gd^3+^) differs with the *e/a* (see the inset of Figure 3). By reducing the *e/a*, *H*_sat._ reaches a minimum of ∼1 T at *e/a* ≈ 1.70 rising again by a further decrease in *e/a*. A lower *H*_sat._ means that the system requires a smaller external magnetic field to fully align the spins, indicating stronger intrinsic magnetic interactions. This aligns well with the fact that both θ_w_ and *T*_C_ are maximized at around *e/a* ≈ 1.70.

In the present ACs, a sharp rise in magnetic susceptibility and field-dependent magnetization, both indicative of the establishment of FM order, are observed across the *e/a* range of 1.60(1)–1.83(1) with the borders corresponding to Ga_28_Au_33_Pt_25_Gd_14_ (*e/a* ≈ 1.60) and Ga_40_Au_21_Pt_25_Gd_14_ (*e/a* ≈ 1.83) 1/1 ACs. To further confirm the presence of FM order in this range, zero-field specific heat (*C*_p_) is measured for three samples, one near the center and two near the borders, as shown in Figure 4. Clearly, all showcase a pronounced anomalies near *T*_C_.

**Figure 4:**
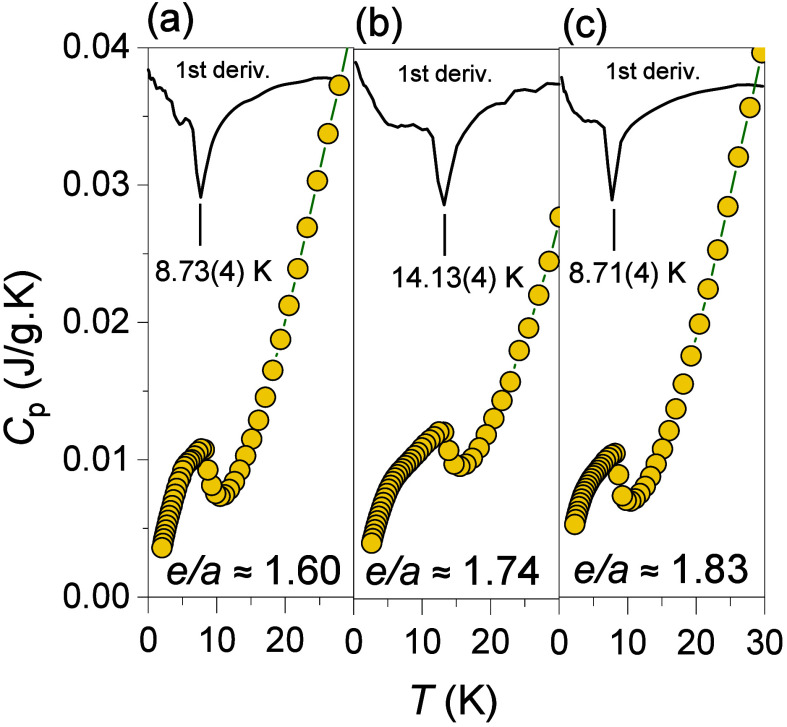
Temperature dependence of *C*_p_ for (a) Ga_28_Au_33_Pt_25_Gd_14_ (*e/a* = 1.60), (b) Ga_33_Au_33_Pt_20_Gd_14_ (*e/a* = 1.74), and (c) Ga_40_Au_21_Pt_25_Gd_14_ (*e/a* = 1.83) 1/1 ACs under a 0 T magnetic field.

### 3 Critical Behavior near *T*_C_

To determine the nature of the magnetic phase transition, the *M* ^2^ dependence of μ_0_(*H*/*M*) in the form of a *standard* Arrott plot^33^ is investigated for four distinct FM 1/1 ACs with varying *e/a* ratios of ≈1.60, 1.71, 1.75, and 1.83 (see Figure S4 of the Supporting Information). The absence of a negative slope and/or an inflection point in the Arrott plot is indicative of a second-order phase transition based on the Banerjee criterion.^34^ According to the scaling principle, in the second-order phase transition the following relations should hold near *T*_C_:^35^

1 $$M_{s}\left(T\right)=M_{0}{\left(-\epsilon \right)}^{\beta };\epsilon <0;T<T_{C}$$

2 $${\left(H/M\right)}_{0}\left(T\right)=\left(h_{0}/M_{0}\right)\epsilon^{\gamma };\epsilon >0;T>T_{C}$$

where *M*_0_ and *h*_0_ are critical amplitudes and ϵ is the reduced temperature (*T – T*_C_)/*T*_C_. The critical exponents β and γ correspond to the spontaneous magnetization *M*_*s*_(*T*) below *T*_C_(*H* = 0) and initial inverse magnetic susceptibility (*H*/*M*)_0_(*T*) above *T*_C_, respectively. Following the approach discussed in the Supporting Information and Figure S5 within, the β and γ within a range of 0.43–0.50 and 0.97–1.03, respectively, are derived (see Table 2 for the values). Note that the approximate errors in the estimation of β, γ, and δ are ±0.05, ±0.1, and ±0.3, respectively. Accordingly, *modified* Arrott plots are constructed, as provided in Figure 5 for Ga_30_Au_33_Pt_23_Gd_14_ 1/1 AC, as an example. Modified Arrott plots for other FM samples are provided in Supplemental Figure S6. Nearly parallel isotherms in modified Arrott plots within a magnetic field range of 0.4–2.5 T (colored sections) with the one close to *T*_C_ passing through the origin confirm the credibility of the adopted critical exponents. Using Widom’s identity,^36^ the estimated δ = 1 + γ/β becomes within 2.92–3.39, which are significantly lower than those expected for three-dimensional (3D) universality classes: δ = 4.80 ^33^ (3D Heisenberg), δ = 4.82 ^33^ (3D Ising), and δ = 5.00 ^34^ (tricritical mean-field) but fairly close to the *γ*/*β* = 3.00 predicted by the Landau mean-field model^33^ (see Table 2). This indicates a mean-field nature of the newly developed FM 1/1 ACs near their *T*_C_ across a wide *e/a* space spanning from ≈1.60 to 1.83.

**Figure 5:**
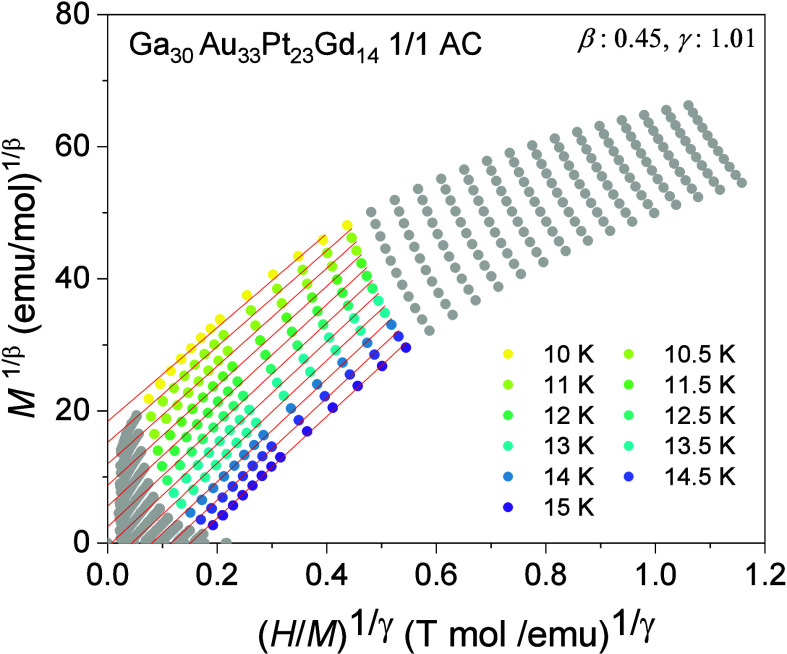
Modified Arrott isotherms in a form of *M* ^1/β^ vs *H*/*M* ^1/γ^ for Ga_30_Au_33_Pt_23_Gd_14_ 1/1 AC. Nearly parallel linear fittings can be observed within the colored sections of the isotherms corresponding to magnetic fields of 0.4 *T* < *H* < 2.5 T.

**Table 2:** Comparison of Critical Exponents (*β*, *γ*, and *δ*) and *T*_C_ for the Present Ga-Pt-Au-Gd FM 1/1 ACs^a^

Composition	β	γ	δ	*T*_C_ (K)	Reference
Ga_40_Au_21_Pt_25_Gd_14_	0.50	0.97	2.94	8.8	This work
Ga_30_Au_33_Pt_23_Gd_14_	0.45	1.01	3.24	12.8	This work
Ga_31_Au_35_Pt_20_Gd_14_	0.44	0.99	3.25	14.9	This work
Ga_28_Au_33_Pt_25_Gd_14_	0.43	1.03	3.39	8.8	This work
Mean field	0.50	1.00	3.00	–	^33^
Tricritical Mean Field	0.25	1.00	5.00	–	^34^
3D Ising	0.325	1.24	4.82	–	^33^
3D Heisenberg	0.365	1.386	4.82	–	^33^

a Theoretical values of the critical exponents for the mean field, tricritical mean field, 3D Ising, and 3D Heisenberg are also provided. Note that the approximate errors in the estimation of *β*, *γ*, and *δ* are ±0.05, ±0.1, and ±0.3, respectively.

### 4 Magnetocaloric Effect

Next, we investigated the Δ*S*_M_ of the present ACs around their transition temperatures. For that purpose, series of temperature-dependent FC magnetization curves within a temperature range of 1.8–100 K and magnetic fields up to 7 T are collected for each sample (see Figure S7 of the Supporting Information). The Δ*S*_M_ is estimated using the thermodynamic Maxwell relation:^37^

3 $$\Delta S_{M}\left(T,H\right)=\mu_{0}\int_{0}^{H_{\max}}{\left(\frac{\partial M\left(T,H\right)}{\partial T}\right)}_{H}dH$$

where *M* and *H* represent the magnetization and the external magnetic field, respectively. In this study, *H*_max_ corresponds to 7 T. In all ACs, −Δ*S*_M_ exhibits a maximum around the transition temperature. The magnitude of the peak in −Δ*S*_M_ at each magnetic field is collected from Figure S7. Based on the collected −Δ*S*_M_ data set, a colormap of Δ*S*_M_ versus *e*/*a* is generated, as shown in Figure 6. Note that the uncertainty in the estimated Δ*S*_M_ values arising from the numerical differentiation and integration in eq 3 is approximately ±0.17 J/ K mol-Gd. In the colormap, the left and right vertical axes are μ_0_ *H* and transition temperature, *T*_C_ or *T*_f_ (freezing temperature in the spin-glass samples), depending on the *e/a*, respectively, with the background color representing the magnitude of |Δ*S*_M_|. The *T*_C_ or *T*_f_ is estimated from the first derivative of ZFC curves, as shown on top of each panel in Figure 2 with an approximate error of ±0.04 K. Black dots in the colormap represent the original data set from which the colormap is constructed.

**Figure 6:**
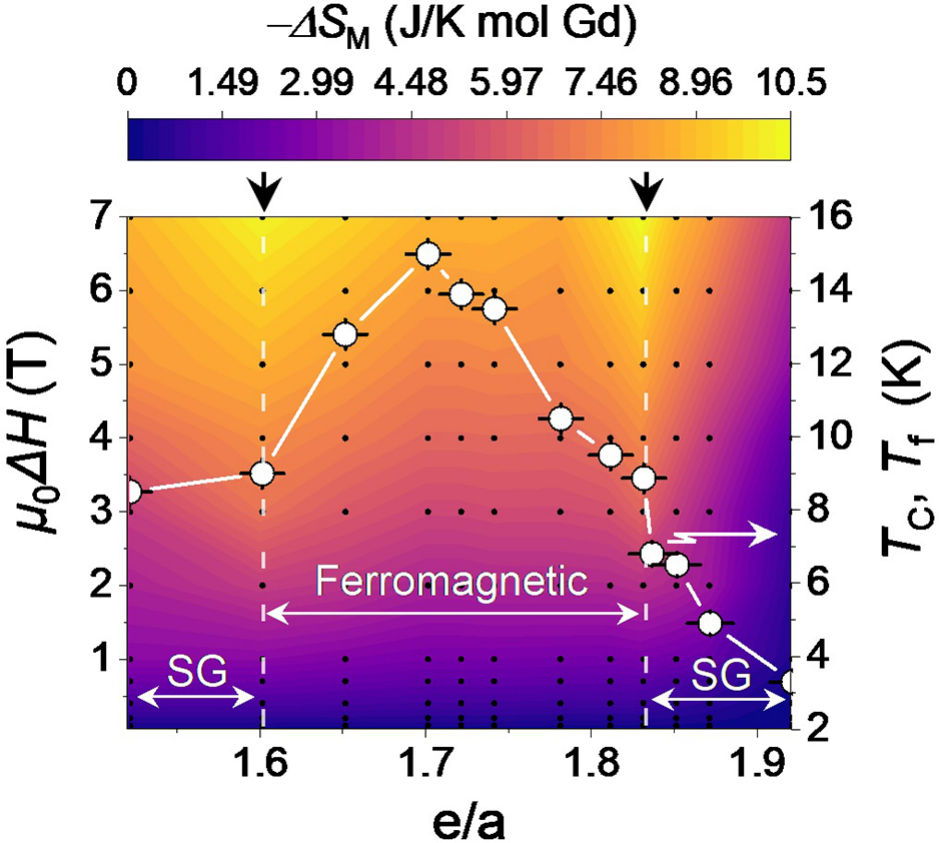
Map of −Δ*S*_M_ versus μ_0_ *H* and the *e*/*a* ratio. The variation of Curie temperature, *T*_C_, estimated from the minimum of the *d*(*M*/*H*)/*dT* curves and freezing temperature (*T*_f_) in spin-glass (SG) samples estimated from the bifurcation point of FC and ZFC curves are also coplotted (white dots). Black dots in the colormap represent the original data set from which the colormap is constructed. The uncertainty in the estimated Δ*S*_m_ values arising from the numerical differentiation and integration in eq 3 is approximately ±0.17 J/ K mol-Gd.

The Δ*S*_M_ colormap in Figure 6 is informative from several aspects: first, it displays two pronounced maxima for the |Δ*S*_M_| at *e*/*a* ∼ 1.60 and 1.83, corresponding to the boundaries of the FM region in the *e*/*a* parameter space. In other words, the magnetic refrigeration response is maximized at particular *e*/*a* values. At *e*/*a* ∼ 1.83, for instance, the −Δ*S*_M_ reaches −8.7 J/K mol-Gd under μ_0_Δ*H* = 5 T (or 10.35 J/K mol-Gd under μ_0_Δ*H* = 7 T), marking the highest |*ΔS*_M_| value ever reported among QCs and ACs. The presence of two maxima in −Δ*S*_M_ at the borders of the FM region where *T*_c_ is minimum reflects an inverse correlation between Δ*S*_M_ and *T*_c_, as commonly seen in R compounds with dominant Ruderman–Kittel–Kasuya–Yosida (RKKY) interaction.^38^ The inverse correlation often follows Δ*S*_M_ ∝ *T*_C_^–2/3^ derived from the Weiss mean field equation of state and the Taylor expansion of the Brillouin function (*B*_J_)^39^ expressed as

4 $$\begin{array}{c} -\Delta S_{M}\left(T,\mu_{0}H\right)=\frac{1}{2C}{\left(\frac{C^{2}}{K}\right)}^{2/3}{\left(\frac{\mu_{0}H}{T_{C}}\right)}^{2/3}+... \end{array}$$

where *C* and *K* are constants depending on the total angular momentum. According to eq 4, the field-dependent magnetic entropy change at *T*_C_, i.e., Δ*S*_M_(*T*_C_, μ_0_ *H*), scales with μ_0_ *H*/*T*_C_ for the given R element, thus inevitably decreasing by increasing *T*_C_.

To gain a comprehensive perspective, −Δ*S*_M_ (in units of J/K mol-R) versus *T*_C_ is plotted in Figure 7 for the present Ga-Au-Pt-Gd 1/1 ACs along with other ACs and heavy R-based compounds reported elsewhere^38^ under μ_0_Δ*H* = 5 T (a field typically generated by commercial superconducting magnets^40^). The |Δ*S*_M_| of the present Ga-Au-Pt-Gd 1/1 ACs (represented by red markers) appear at the far-left side of Figure 7 where the highest Δ*S*_M_ values are expected based on eq 4. Clearly, the present Ga-Au-Pt-Gd 1/1 ACs not only outperform previous Au-based ACs^41^ by 33% in Δ*S*_M_ magnitude but also showcase comparable Δ*S*_M_ values to other high-performance heavy R-based compounds, such as RCo_2_ ^42^ and Er_5_Si_4_.^43^

**Figure 7:**
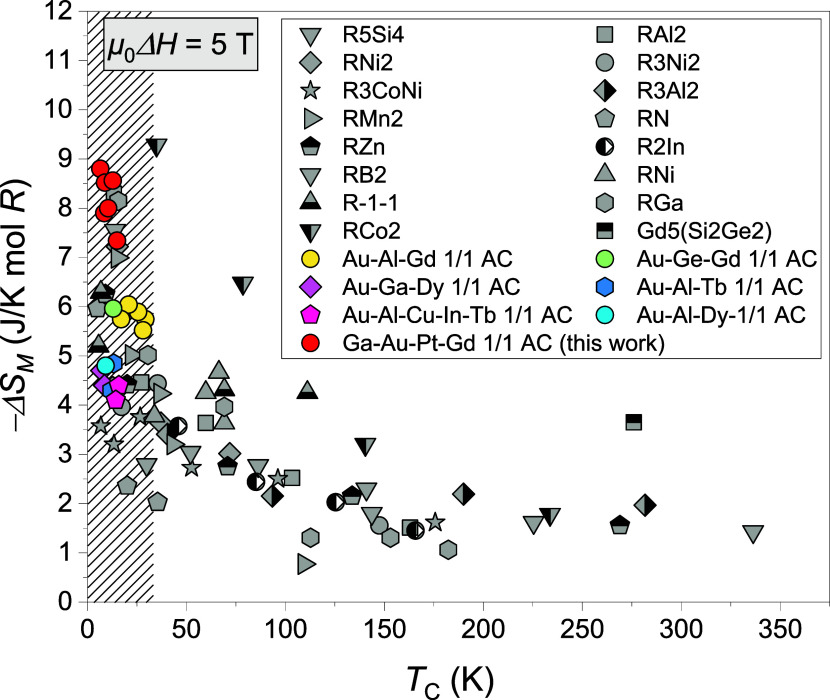
*T*_C_ dependence of −Δ*S*_M_ of the present Ga-Pt-Au-Gd 1/1 ACs and several Au-based ACs as well as other heavy R-based compounds under the magnetic field change of 5 T. The unit of −Δ*S*_M_ is J/K mol-R (per 1 mol of R atoms). The shaded part at the low-temperature region indicates the *T*_C_ range accessible in Tsai-type QCs and ACs. The uncertainty in the estimated Δ*S*_M_ values, primarily arising from the numerical differentiation and integration procedure in eq 3, is approximately ±0.17 J/ K mol-Gd, which is smaller than the symbol size.

To further assess magnetocaloric effect performance, the relative cooling power (RCP), a measure of heat transfer between the hot and cold reservoirs, and the temperature-averaged entropy change (TEC)^44^ are calculated for the FM samples across a wide *e*/*a* range of 1.60–1.83 using

5 $$RCP={\Delta S}_{\max}\times {\delta T}_{FWHM}$$

and

6 $$TEC\left(\Delta T\right)=\frac{1}{\Delta T}\max\left\{\int_{T_{mid}-\Delta T/2}^{T_{mid}+\Delta T/2}\left|{\Delta S}_{M}\left(T\right)\right|dT\right\}$$

respectively. In eq 5, δ*T*_FWHM_ denotes the full width at half-maximum of the corresponding Δ*S*_M_(*T*) curve. In eq 6, the temperature range Δ*T* is set to 5 K and *T*_*mid*_ is chosen by sweeping over the available Δ*S*_M_(*T*) to find the best value that maximizes TEC. The results, as shown in Figure S8­(a) and (b), evidence an RCP value of ∼163 J/kg under μ_0_Δ*H* = 5 T. Likewise, under a 5 T field change, the TEC values exhibit a dip at *e*/*a* = 1.70 and two maxima at *e*/*a* ≈ 1.60 and 1.83, in accordance with the trend observed in Figure 6.

Furthermore, adiabatic temperature change (*ΔT*_ad_), another parameter for assessing magnetocaloric effect performance alongside Δ*S*_M_, is calculated under varying magnetic fields for the two 1/1 ACs with the highest Δ*S*_M_ values [located at *e*/*a* ≈ 1.60 and 1.83, as marked by arrows in Figure 6]. The results (shown in Figure 8c of the Supporting Information) evidence a power law correlation between Δ*T*_ad_ and the applied magnetic field (Δ*T*_ad_ ∝ *H* ^*n*^), with fitted exponent of *n* being in a range of 0.66–0.68, which are fairly close to the exponent 2/3 predicted by mean-field approximation. This is consistent with the mean-field critical behavior of the present 1/1 ACs revealed in Section 3.

Lastly, the approach introduced in this work shows high potential for turning *stoichiometric* magnetically frustrated compounds into *nonstoichiometric* magnetically ordered systems with outstanding magnetic entropy response. This is certainly a significant step forward in materials design that could open new opportunities for developing high-performance magnetocaloric materials. A key advantage of this approach is that it is not restricted to the specific alloy family and, in principle, can be expanded to other alloy systems. Note that the present study particularly benefits from high miscibility between Au and Pt, which share similar atomic radii (Au: 144 pm, Pt: 139 pm),^45^ comparable electronegativities (Au: 2.54, Pt: 2.28, Pauling scale^46^), and close valence electron numbers (Au: 1, Pt: 0). These comparable properties promote solid-solution formation by effectively reducing the structural strain upon substitution. Therefore, this approach is particularly effective when substituting elements with a high mutual miscibility. Depending on the desired *e*/*a* ratio and the constituent elements of the target alloy, other elemental pairs such as Cu/Mg, Ca/Pb, or Ag/Pd could also be considered for substitution. Moreover, this approach could contribute to reducing synthesis costs by allowing substitution of expensive elements with more affordable alternatives such as Cu and Ag, which is definitely an important consideration in magnetocaloric technology.

## Conclusion

In summary, we present a novel materials design strategy called a “double heterovalent elemental substitution” that overcomes the compositional limitation of stoichiometric intermetallic compounds by breaking the structural constraints and expanding the compositional degrees of freedom across a broad valence electron-per-atom (*e/a*) parameter space. By employing this strategy in the stoichiometric Ga-Pt-Gd 2/1 approximant crystals (ACs), we demonstrated the emergence of a new family of nonstoichiometric 1/1 ACs exhibiting long-range ferromagnetic (FM) order with mean-field-like critical behavior. Most strikingly, the isothermal magnetic entropy change (Δ*S*_M_) reached a value of −8.7 J/K mol-Gd under a 5 T field change at particular *e/a* values, surpassing all previously reported Δ*S*_M_ values for quasicrystals and ACs. Given the transition temperature range of 8.7–14.9 K in the present ACs, these materials may be considered for potential applications in adiabatic cooling or as passive regenerators^37^ that are used for cooling systems down to the sub-Kelvin regime. Our findings provide a new material design strategy applicable to any stoichiometric compound with a potential for designing high-performance magnetocaloric materials.

## Experiments

The synthesis protocol includes arc-melting of the constituent elements under an argon atmosphere. Each sample was melted three times to ensure homogeneity. Following arc-melting, the samples were annealed at 1073 K for 50 h in an argon-filled quartz tube to homogenize the microstructure. The phase purity of the samples after synthesis was confirmed through powder X-ray diffraction (XRD) using a Rigaku SmartLab SE X-ray diffractometer with Cu–Kα radiation. A representative sample with a nominal composition of Ga_33_Au_33_Pt_20_Gd_14_ was selected for a room temperature single-crystal X-ray diffraction (SCXRD) experiment using an XtaLAB Synergy-R single-crystal diffractometer equipped with a hybrid pixel array detector (HyPix6000, Rigaku) with Mo Kα radiation (λ = 0.71073 Å).

For bulk magnetization measurement, a superconducting quantum interference device (SQUID) magnetometer (Quantum Design, MPMS3) was utilized under zero-field-cooled (ZFC) and field-cooled (FC) modes within a temperature range of 1.8 to 300 K by applying external dc fields up to 7 × 10^4^ Oe. Additionally, ac magnetic susceptibility measurements were carried out at frequencies ranging from 0.1 to 100 Hz within a temperature range of 2–20 K and ac magnetic field amplitude (*H*_ac_) of 1 Oe. Specific heat measurements were conducted in a temperature range of 2–40 K by a thermal relaxation method using a Quantum Design Physical Property Measurement System (PPMS). The Δ*S*_M_ values of the samples are derived from isothermal magnetization measurements conducted up to μ_0_ *H* = 7 T at various temperatures using the thermodynamic Maxwell relation.

## Supplementary Material



## Notes

Reconstructed reciprocal-space sections from single-crystal X-ray diffraction; temperature dependence of inverse magnetic susceptibility *a*; ac susceptibility in-phase component across frequencies and compositions; Arrott plot in the form of *H*/*M* vs *M* ^2^ for ferromagnetic samples; scaling analysis of the phase transition near Curie temperature in ferromagnetic samples; modified Arrott isotherms in the form of *M* ^1/β^ vs *H*/*M* ^1/γ^ with β and γ corresponding to critical exponents; series of temperature-dependent magnetic entropy change for ferromagnetic samples under field-cooled conditions; relative cooling power, temperature-averaged entropy change, and adiabatic temperature change as a function of applied field for selected compositions (PDF)
