# Supplementary material for: Derivation of a Nonstoichiometric 1/1 Quasicrystal Approximant from a Stoichiometric 2/1 Quasicrystal Approximant and Maximization of the Magnetocaloric Effect
Source: J Am Chem Soc. 2025 Aug 27;147(37):33424–31. doi: 10.1021/jacs.5c05947 (PMC12447484; doi:10.1021/jacs.5c05947)
Supplement: Supplementary file 1 [file ja5c05947_si_001.pdf]

Supplemental Material

Derivation of a Nonstoichiometric 1/1 Quasicrystal Approximant from a Stoichiometric 2/1 Quasicrystal Approximant and Maximization of the Magnetocaloric Effect

Farid Labib[a],* Hiroyuki Takakura[b], Asuka Ishikawa[a], Takenori Fujii[c], Ryuji Tamura[d],*

[a] *Research Institute of Science and Technology, Tokyo University of Science, Tokyo 125-8585, Japan*

[b] *Division of Applied Physics, Faculty of Engineering, Hokkaido University, Sapporo 060-8628, Japan*

[c] *Cryogenic Research Center, The University of Tokyo, Bunkyo, Tokyo 113-0032, Japan*

[d] *Department of Materials Science and Technology, Tokyo University of Science, Tokyo 125-8585, Japan*

Corresponding authors:
labib.farid@rs.tus.ac.jp
tamura@rs.tus.ac.jp

Table of contents

Figure S1 displays reconstructed reciprocal-space sections along main zone axes of (a) [100], (b) [110] and (c) [111] obtained from single crystal X-ray diffraction (SCXRD) data derived from $Ga_{33}Au_{33}Pt_{20}Gd_{14}$ 1/1 AC.

Figure S2 shows temperature-dependence of inverse magnetic susceptibility (*H/M*) of the studied samples in the paper under $\mu_0 H = 0.1$ T within a temperature range of 1.8–300 K. The inset shows electron-per-atom (*e/a*) dependence of the Θ_w/dG (*dG* denotes de Gennes parameter). The curve in the inset is a polynomial fitting to the Θ_p/dG values.

Figure S3 shows in-phase component of ac magnetic susceptibility (χ'_{ac}) for the samples studied in the paper under *f* spanning three orders of magnitude from 0.1 to 100 Hz.

Figure S4 shows Arrott plot in the form of *H/M* vs. M^2 for (a) $Ga_{28}Au_{33}Pt_{25}Gd_{14}$, (b) $Ga_{30}Au_{33}Pt_{23}Gd_{14}$, (c) $Ga_{31}Au_{35}Pt_{20}Gd_{14}$ and (d) $Ga_{40}Au_{21}Pt_{25}Gd_{14}$ 1/1 ACs. Based on the Banerjee's criterion, the absence of a clear negative slope or inflection point indicate a second-order nature of the phase transitions.

Figure S5 coplots temperature dependence of spontaneous magnetization M_S *(T)* (left axis) and initial inverse magnetic susceptibility $(H/M)_0$ (right axis) for (a) $Ga_{28}Au_{33}Pt_{25}Gd_{14}$, (b) $Ga_{31}Au_{35}Pt_{20}Gd_{14}$ (c) $Ga_{30}Au_{33}Pt_{23}Gd_{14}$ and (d) $Ga_{40}Au_{21}Pt_{25}Gd_{14}$ 1/1 ACs. Based on the scaling principle, in the second-order phase transition, the equations (1) and (2) in the main text should hold near the critical temperature (T_C). The lines in Fig. S5 indicate the fittings to equations (1) and (2) in the main text, from which the derived critical exponents β and γ are derived. The range of β and γ is within 0.43 – 0.50 and 0.97 – 1.03, respectively. The critical exponent β is correlated with the spontaneous magnetization $M_s(T)$ at $H = 0$ below T_C, while γ corresponds to initial inverse magnetic susceptibility $(H/M)_0(T)$ above T_C. The initial values of M_s and $(H/M)_0$ is attained from linear extrapolation of the modified Arrott-plot isotherms and their interceptions with the $M^{1/\beta}$ and $(H/M)^{1/\gamma}$ axes (i.e., below and above T_C), respectively. Here, the iteration approach is applied meaning that the β and γ values are estimated from fittings to the equations (1) and (2) in the main text, respectively. Then, the estimated critical components are used to reconstruct new modified Arrott plots from which newer sets of critical exponents are extracted. The above process continues until a convergence in the fitting values is obtained meaning that they become independent from the initial parameters and reflect the intrinsic behavior of the material.

Figure S6 shows modified Arrott isotherms in a form of $M^{1/\beta}$ vs. $H/M^{1/\gamma}$ for (a) $Ga_{28}Au_{33}Pt_{25}Gd_{14}$ 1/1 AC, (b) $Ga_{31}Au_{35}Pt_{20}Gd_{14}$ 1/1 AC and (c) $Ga_{40}Au_{21}Pt_{25}Gd_{14}$ 1/1 AC. Nearly parallel linear fittings can be observed within the colored sections of the isotherms corresponding to magnetic fields of 0.4 T < *H* < 2.5 T.

Figure S7 shows series of temperature dependent ΔS_M curves under field-cooled (FC) condition and magnetic field spanning from 0.01 – 7 T for (a) $Ga_{52}Pt_{34}Gd_{14}$ 2/1 AC, (b) $Ga_{46}Au_7Pt_{33}Gd_{14}$, (c) $Ga_{43}Au_{12.5}Pt_{30.5}Gd_{14}$, (d) $Ga_{41}Au_{20}Pt_{25}Gd_{14}$, (e) $Ga_{40}Au_{21}Pt_{25}Gd_{14}$, (f) $Ga_{39}Au_{22}Pt_{25}Gd_{14}$, (g)

$Ga_{37}Au_{25}Pt_{24}Gd_{14}$, (h) $Ga_{33}Au_{33}Pt_{20}Gd_{14}$, (i) $Ga_{33}Au_{31}Pt_{24}Gd_{14}$, (j) $Ga_{30}Au_{33}Pt_{23}Gd_{14}$, (k) $Ga_{28}Au_{33}Pt_{25}Gd_{14}$, and (l) $Ga_{25}Au_{35}Pt_{26}Gd_{14}$ 1/1 ACs.

Figure S8 shows (a) relative cooling power (RCP) and (b) temperature averaged entropy change (TEC) parameters calculated for the FM samples with *e/a* values in a range of 1.60 – 1.83 using the equations (5) and (6) in the main text, respectively. (c) shows variation of adiabatic temperature change (ΔT_{ad}) with the applied magnetic field (H) for the two samples with nominal compositions of $Ga_{28}Au_{33}Pt_{25}Gd_{14}$ (*e/a* = 1.60), and $Ga_{40}Au_{21}Pt_{25}Gd_{14}$ (*e/a* = 1.83).

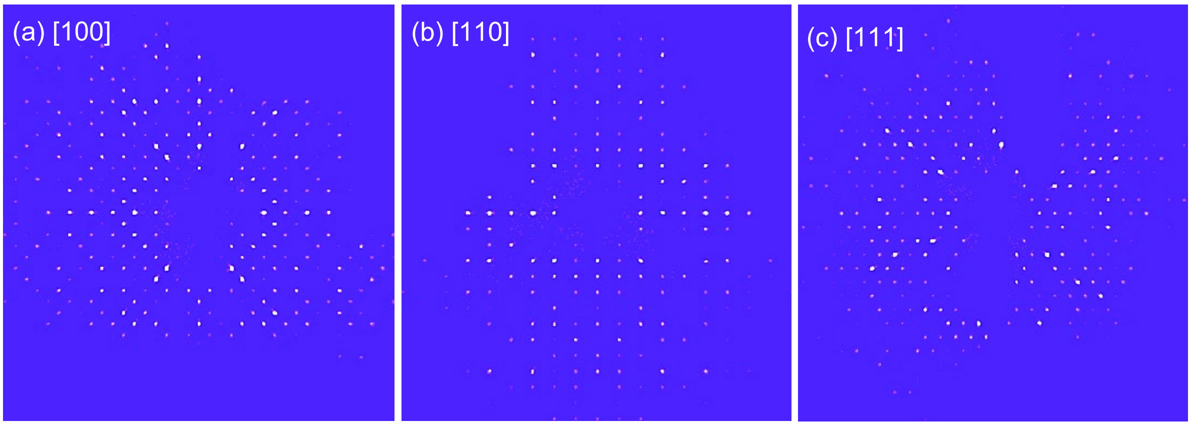

Figure S1. Reconstructed reciprocal-space sections derived from single crystal X-ray diffraction (SCXRD) data taken from $Ga_{33}Au_{33}Pt_{20}Gd_{14}$ 1/1 AC along main zone axes of (a) [100], (b) [110] and (c) [111].

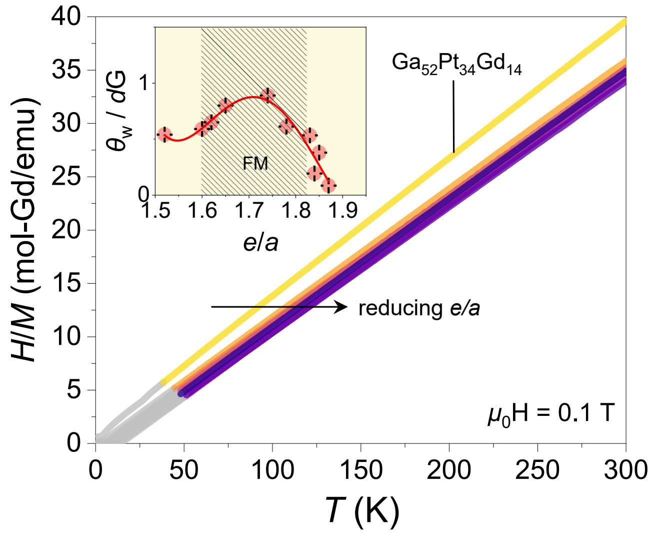

Figure S2. Temperature-dependence of inverse magnetic susceptibility (*H/M*) of the samples under $\mu_0 H = 0.1$ T within a temperature range of 1.8–300 K. The inset shows electron-per-atom (*e/a*) dependence of the Θ_w/dG for the present Ga-based samples. The curve in the inset is a polynomial fitting to the Θ_w/dG values (Θ_w and dG correspond to Curie-Weiss temperature and de Gennes parameter, respectively). The error bars in the inset, which are comparable to symbol size, represent the uncertainties in both *e/a* (±0.01) and the θ_w/dG values. For the latter, the error bar stems from standard deviations in the linear fits to the data over different temperature ranges.

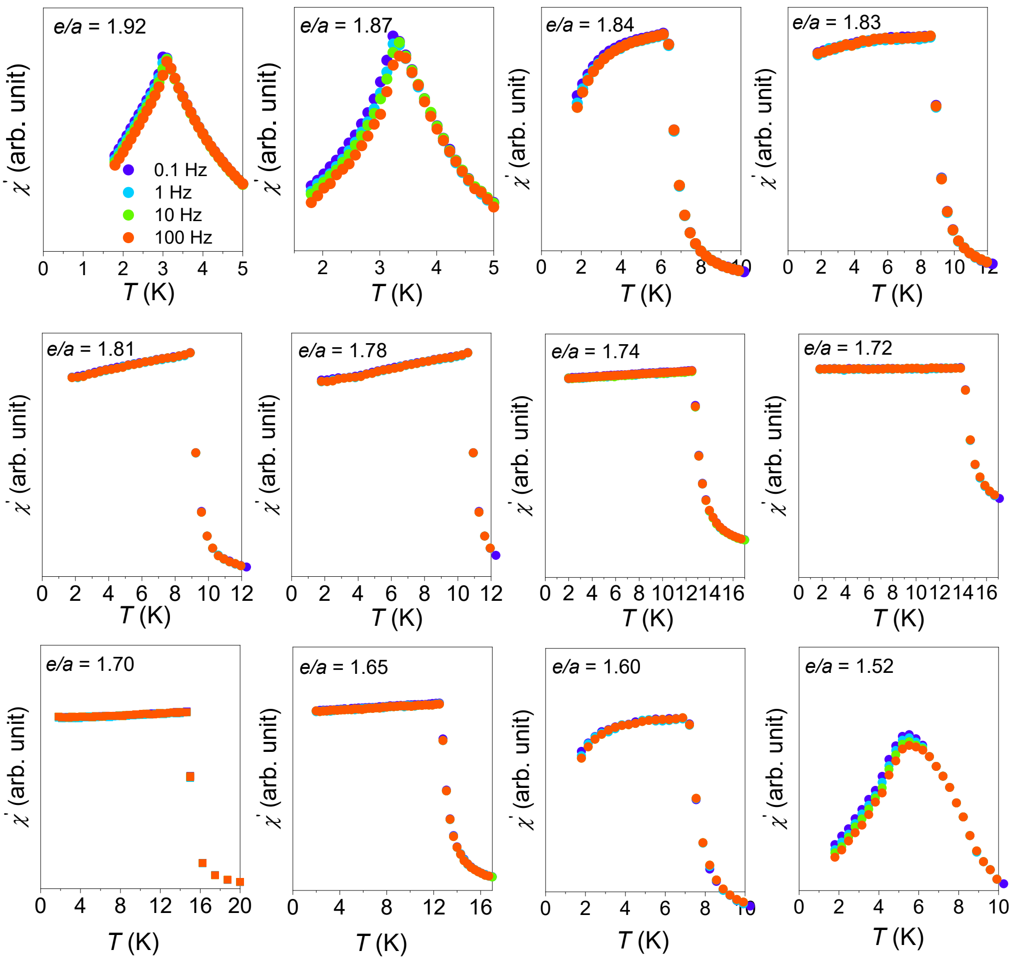

Figure. S3. In-phase component of ac magnetic susceptibility (χ'_{ac}) for 1/1 ACs with varying e/a parameters under *f* spanning three orders of magnitude from 0.1 to 100 Hz.

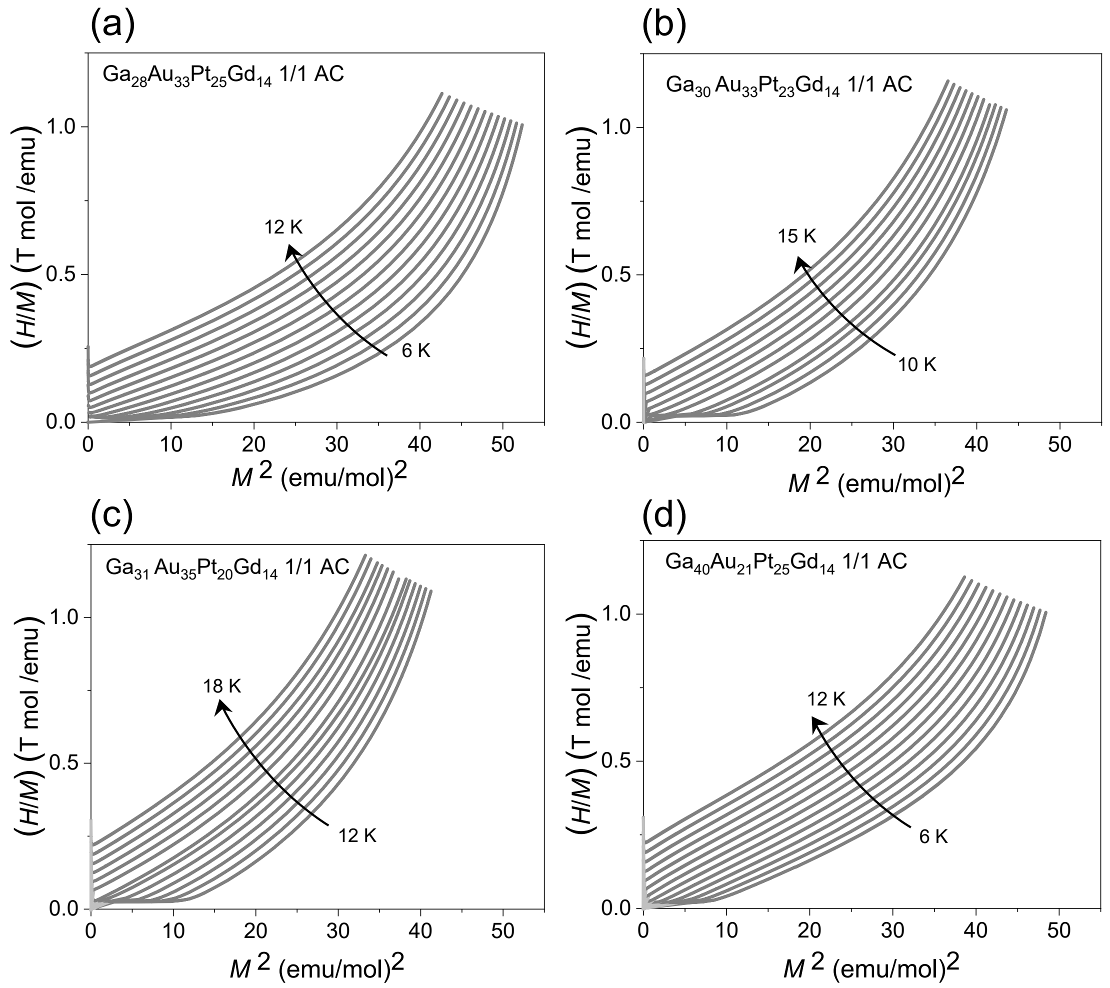

Figure S4. Arrott plots (*H/M* vs. M^2) for (a) $Ga_{28}Au_{33}Pt_{25}Gd_{14}$ 1/1 AC, (b) $Ga_{30}Au_{33}Pt_{23}Gd_{14}$ 1/1 AC, (c) $Ga_{31}Au_{35}Pt_{20}Gd_{14}$ 1/1 AC and (d) $Ga_{40}Au_{21}Pt_{25}Gd_{14}$ 1/1 AC within a temperature range near corresponding Tc.

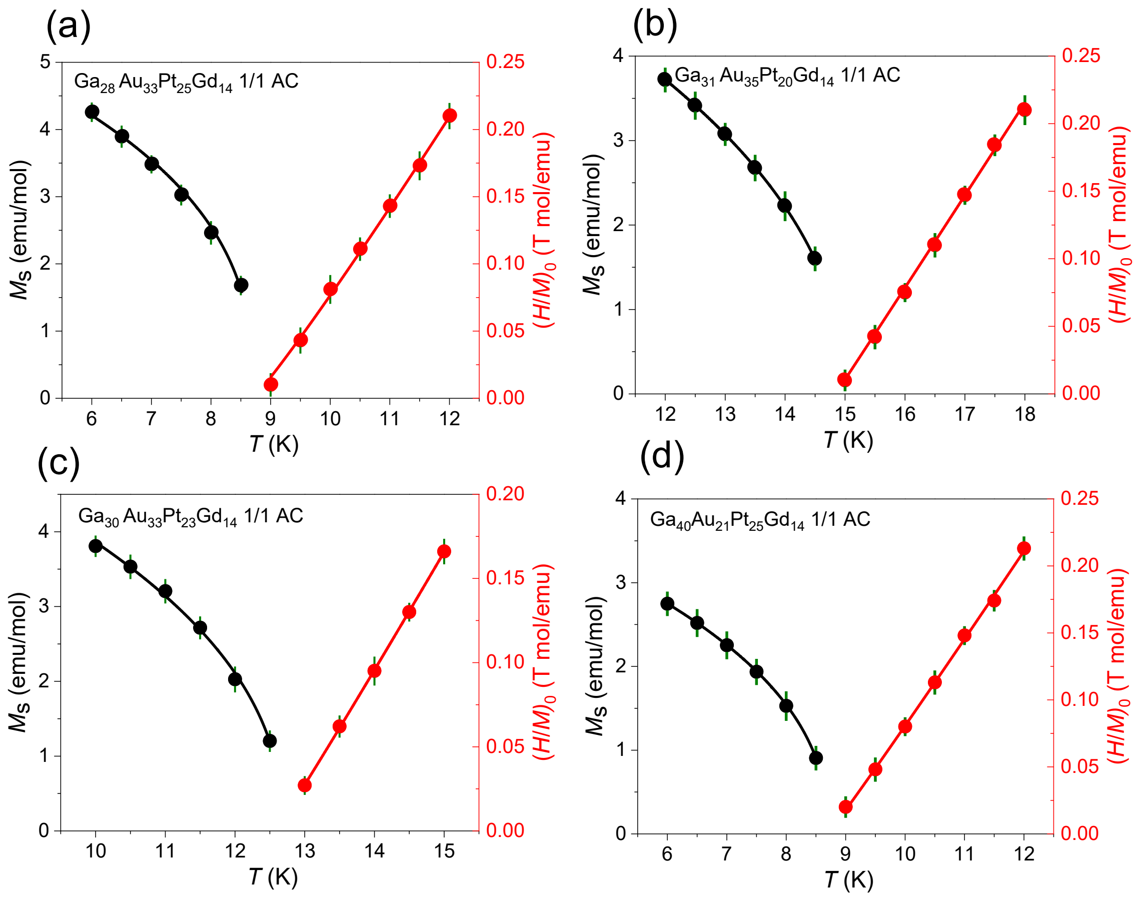

Figure S5. The temperature dependence of M_S *(T)* (left axis) and $(H/M)_0$ (right axis) for (a) $Ga_{28}Au_{33}Pt_{25}Gd_{14}$, (b) $Ga_{31}Au_{35}Pt_{20}Gd_{14}$ (c) $Ga_{30}Au_{33}Pt_{23}Gd_{14}$ and (d) $Ga_{40}Au_{21}Pt_{25}Gd_{14}$ 1/1 ACs. The lines indicate the fitting curves to equations (1) and (2) in the main text. Error bars represent the statistical uncertainty of the linear extrapolation in the modified Arrott plots.

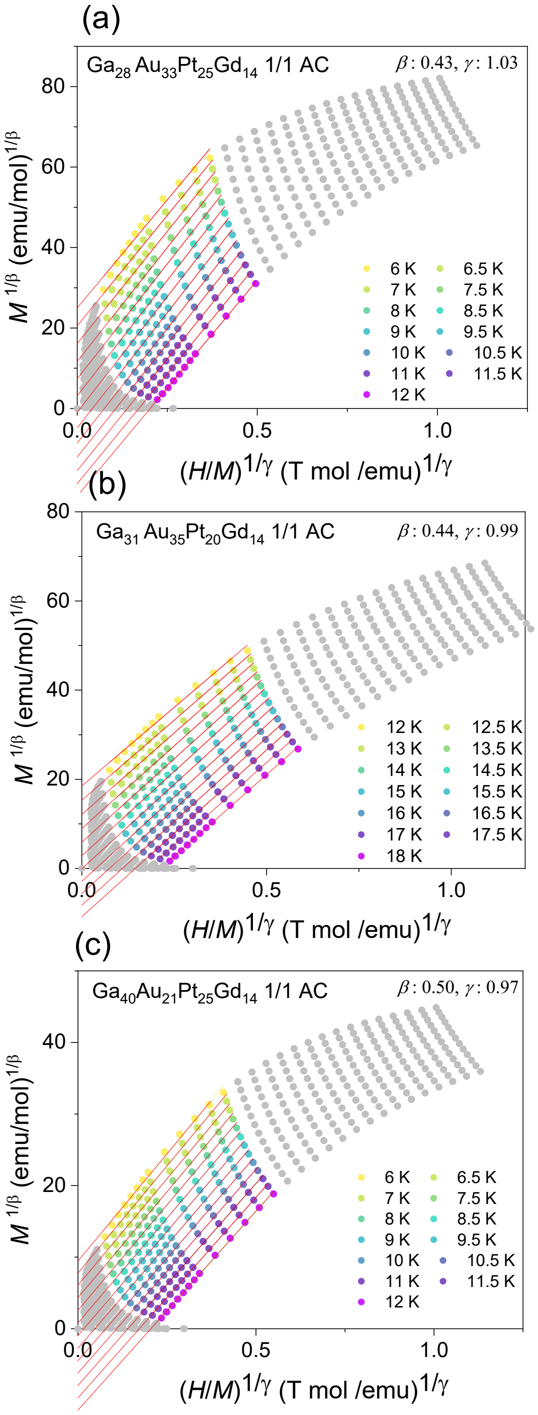

Figure S6. The modified Arrott isotherms in a form of $M^{1/\beta}$ vs. $H/M^{1/\gamma}$ for (a) $Ga_{28}Au_{33}Pt_{25}Gd_{14}$ 1/1 AC, (b) $Ga_{31}Au_{35}Pt_{20}Gd_{14}$ 1/1 AC and (c) $Ga_{40}Au_{21}Pt_{25}Gd_{14}$ 1/1 AC. Nearly parallel linear fittings can be observed within the colored sections of the isotherms corresponding to magnetic fields of 0.4 T < H < 2.5 T.

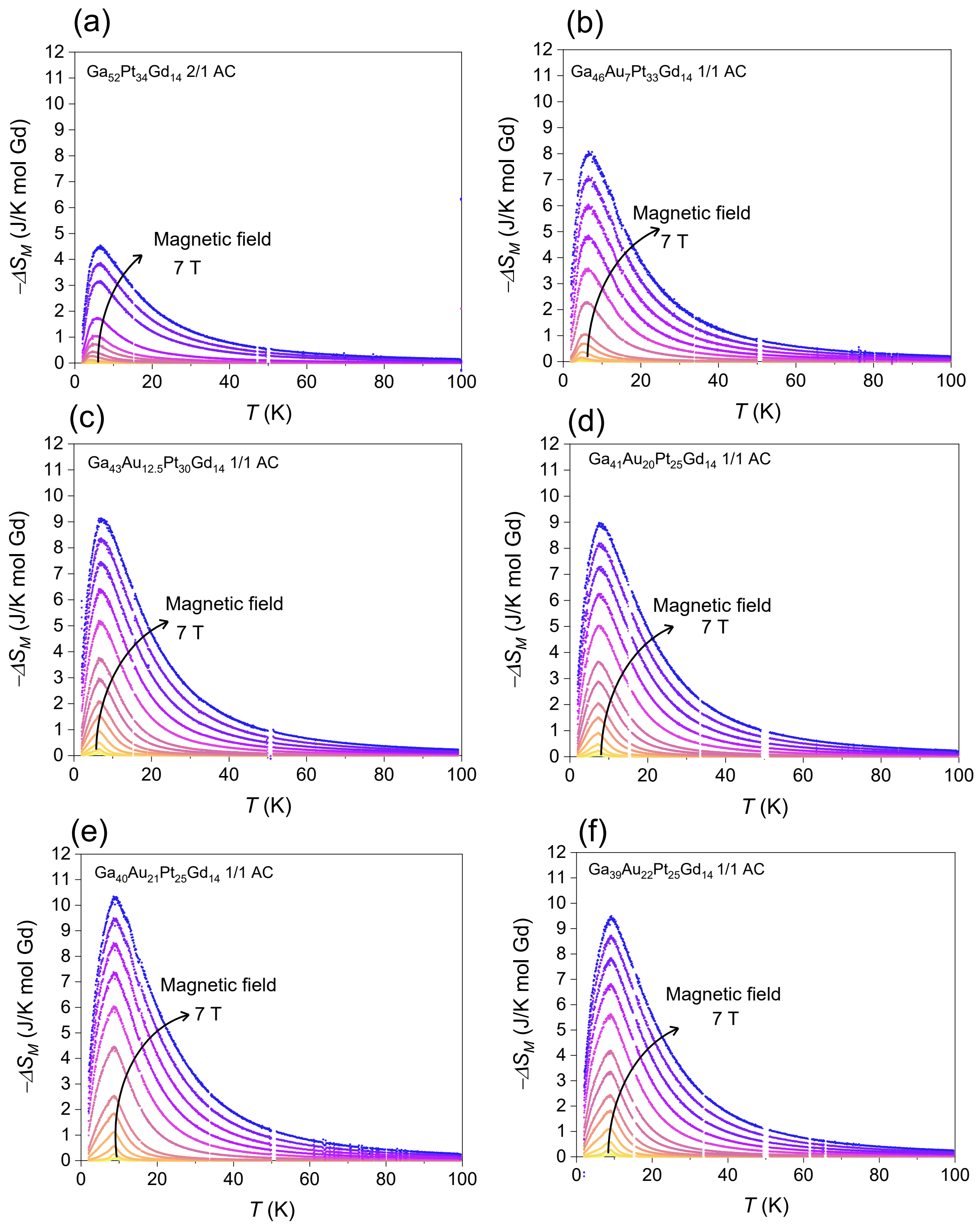

Figure S7. Series of temperature dependence of ΔS_M under FC mode and magnetic field spanning from 0.01 – 7 T for (a) $Ga_{52}Pt_{34}Gd_{14}$ 2/1 AC, (b) $Ga_{46}Au_{7}Pt_{33}Gd_{14}$, (c) $Ga_{43}Au_{12.5}Pt_{30.5}Gd_{14}$, (d) $Ga_{41}Au_{20}Pt_{25}Gd_{14}$, (e) $Ga_{40}Au_{21}Pt_{25}Gd_{14}$, (f) $Ga_{39}Au_{22}Pt_{25}Gd_{14}$, (g) $Ga_{37}Au_{25}Pt_{24}Gd_{14}$, (h) $Ga_{33}Au_{33}Pt_{20}Gd_{14}$, (i) $Ga_{33}Au_{31}Pt_{24}Gd_{14}$, (j) $Ga_{30}Au_{33}Pt_{23}Gd_{14}$, (k) $Ga_{28}Au_{33}Pt_{25}Gd_{14}$, and (l) $Ga_{25}Au_{35}Pt_{26}Gd_{14}$ 1/1 ACs.

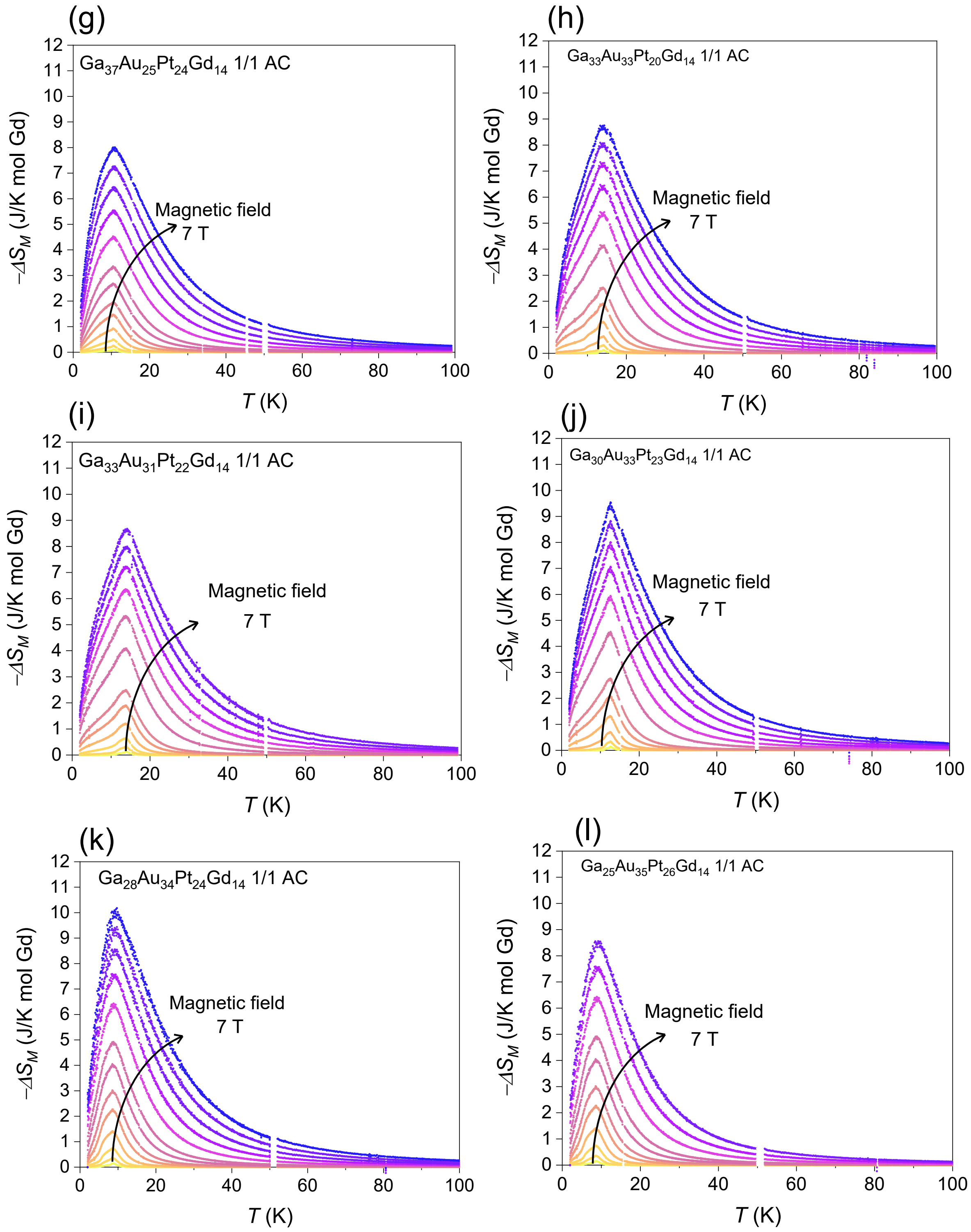

Figure S7. Continued.

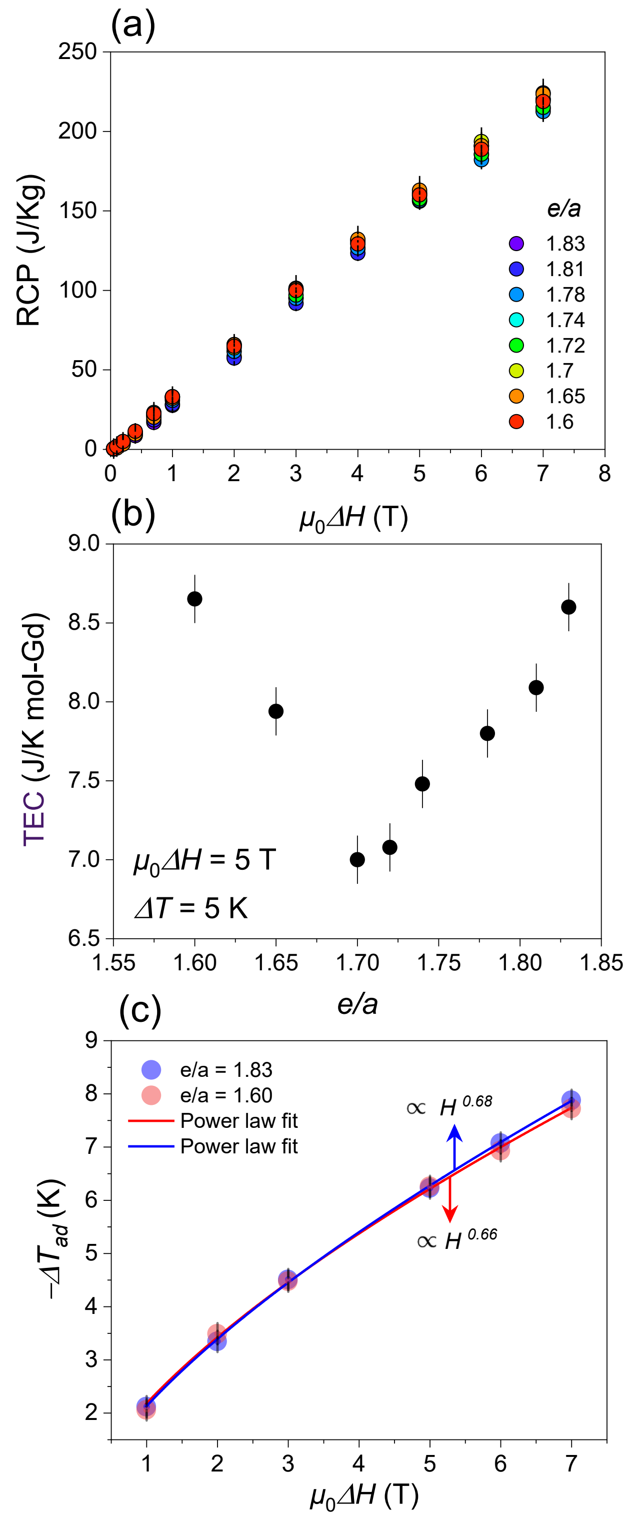

Figure S8. (a) Relative cooling power (RCP) and (b) temperature-averaged entropy change (TEC) parameters calculated for the FM samples with *e/a* values in a range of ≈ 1.60 – 1.83 using the equations (5) and (6) in the main text, respectively. (c) Variation of adiabatic temperature change (ΔT_{ad}) with the applied magnetic field (*H*) for the two samples with composition of $Ga_{28}Au_{33}Pt_{25}Gd_{14}$ (*e/a* ≈ 1.60), and $Ga_{40}Au_{21}Pt_{25}Gd_{14}$ (*e/a* ≈ 1.83). A power law correlation is observed between ΔT_{ad} and *H* ($\Delta T_{ad} \propto H^n$), with fitted exponent *n* within a range of 0.66−0.68, which are close to 2/3 predicted by mean-field approximation. Approximate errors in the estimations are also provided.
